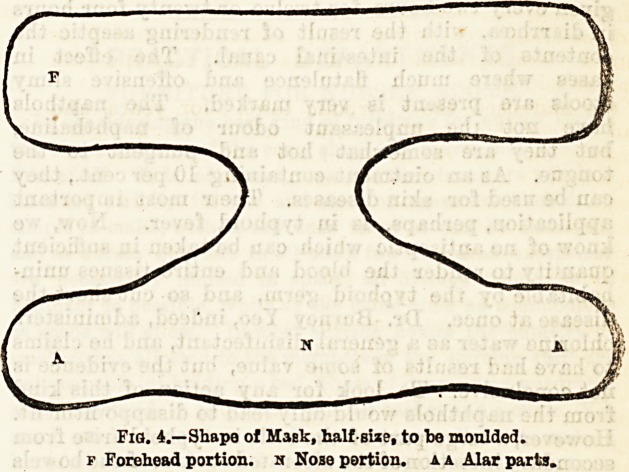# The Treatment of Fractures of the Nose

**Published:** 1893-07-01

**Authors:** Walker Downie

**Affiliations:** Surgeon Throat and Nose Department, Western Infirmary, Glasgow, &c.


### THE TREATMENT OF FRACTURES OF THE NOSE.

By Walker Downie, M.B., F.F.P.S.G., Surgeon Throat and Nose Department, Western Infirmary, Glasgow, &c.

By way of preface it may be stated that the treatment necessary in any given case of fracture of the nose will depend entirely on the portion of the framework of that organ which has been injured, and whether that injury has resulted in the production of deformity or not. Then, again, when deformity has followed a fracture, the means employed to restore the injured feature to its normal shape vary according to whether it is a recent injury or one of long standing which has to be dealt with. Fractures of the nose, though produced in a variety of ways and by various means, are always the result of direct violence. A blow from the clenched hand during fisticuffs, or from a stick, a cricket-ball, or a stone, from the handle of a crane or a pump, falls from a height, collision with the head of an opponent during a charge or scrimmage at football, are but some of the more common causes related by patients in giving an account of their injuries. Before attempting to remedy matters the injured organ must be carefully examined to ascertain what structures have been injured, and if there is deformity, what parts have been displaced.

Immediately following the receipt of a severe blow, the size, form, and general appearance of the nose may be greatly altered by swelling and ecchymosis from tearing of the soft tissues. Under such circumstances the detection of fracture is often difficult, and to determine with precision what structures are involved is frequently impossible. Profuse hæmorrhage from more or less extensive tearing of the highly vascular lining mucous membrane is often associated with such an injury, and though in the majority of cases it ceases spontaneously through the formation of a clot within the nares, yet occasionally it is so severe that when no surgical help is at hand a patient's life may be placed in danger. To check the hæmorrhage cold may be applied, but it is much more quickly and readily controlled by a properly-adjusted pad introduced through the anterior nares. And here I would observe that as in almost every case the tearing of the lining membrane is confined to the anterior half of the nasal cavity, plugging of the posterior nares is rarely required in cases of bleeding from violence applied externally. To make the necessary pledget we take a strong, smooth, straight probe or cotton-holder, and around its free end firmly coil a sufficiency of absorbent or styptic cotton wool to make a pad of the length and thickness shown in fig. 1. This is readily introduced by a rotatory movement, and when the whole plug is within the naris the probe is withdrawn, and the cotton wool left *in situ*. Additional pressure, when required, may be applied externally after the plug has been introduced by the patient grasping his nose between the thumb and first and second fingers.

Fig. 1.—Pledget of Cotton Wool on Probe, ready for use.

After bleeding has ceased, the nose should be carefully examined to ascertain the character of the injuries. The nasal bones, from their unprotected and prominent position, suffer most frequently, and they may be the only portions of the framework which receive injury. When the blow has been received laterally the lachrymal bone and the nasal process of the superior maxilla on the side struck may be fractured. These same parts as well as the vomer and central plate of the ethmoid may be fractured by a direct blow of a severe type, and with a comparatively narrow instrument. The cartilaginous septum is seldom fractured, but it is frequently dislocated, and looking to its highly elastic nature, its position, and its attachments, such a result is only what might be expected. The deformity occurring as the result of fracture depends on the structures fractured, and on the degree of displacement of the fragments. Where the blow has been received by the two nasal bones equally, they may be both fractured and dislocated backwards, and where such has occurred the distance between the two eyes appears to be increased, and the nasal processes of the superior maxillæ become unduly prominent. When the blow has been dealt laterally, the bones may be displaced to one or other side, and if the blow has been sufficient to bend or dislocate the septum, the line of the nose becomes curved. The injury in other cases may be confined to the cartilaginous septum, which may be dislocated and so much displaced laterally as to considerably narrow or even completely block one naris without altering the contour of the nose; on the other hand, as a result of severe direct violence, the septum may be fractured or dislocated and displaced backwards, in which case we have as a result a distinct depression immediately beyond the extremities of the nasal bones, and a slight tilting up of the tip of the nose.

In the treatment of such injuries dislocation of the septum frequently gives considerable trouble, and that is especially the case where it has been displaced backwards, for there after reduction the dislocation readily recurs. But it is of fractures I wish specially to speak. When bleeding has ceased and the swelling somewhat subdued, the position of the fracture should be carefully ascertained, and, that having been done, the displaced portion should be returned to its normal position. This may be accomplished by several means. Depressed portions are readily raised by the use of dresser's forceps introduced closed and used as a lever; but when the fragments are displaced laterally, it is better to employ forceps after the pattern of those recommended by Mr. Wm. Adams. Both blades may be passed into one naris, or one blade into each naris, or one within a naris and the other applied externally, according to the position of the fragment to be replaced. Where the fracture is limited to one portion, it will be found that on setting the fractured bone it remains set, and gives no further trouble; but where the fracture is comminuted, and especially where it is associated with dislocation of the septum, it will be found necessary to apply some apparatus to support and retain the replaced fragments in position. This can, perhaps, be best accomplished by the use of the splints here figured, which consist of two intra-nasal splints and a mask, or external splint, all made of sheet-lead. The intra-nasal splints are in outline shaped somewhat like an almond, and from the broadest part a wing projects on each side (fig. 2). Before being applied, these wings are bent towards each other, so as to form an arch over one surface (fig. 3); and when in position, the flat portion of the splint lies in contact with the septal cartilage, and the curved wings lie against the inferior turbinated body. Pressure over the septum can be increased by separating the wings out from the body of the splint, and increasing the arch formed by the wings. The external splint, or mask (fig. 4), is of one piece, and consists of a lower part, which is moulded to the form of the nose, the alar portions of which are prolonged on each side to rest on the cheeks and give steadiness to the splint, and an upper part which lies on the forehead, and by which the whole apparatus is readily retained in the middle line, and prevented from becoming displaced downwards. It is covered by a layer of silk or linen.

Fig. 2.—Shape of Intra-nasal Splint, full-size.

Fig. 3.—Same, ready for introduction.

Where, then, we are dealing with a recent fracture the displaced portions of bone are carefully replaced by leverage, &c., after which the intra-nasal splints are cautiously introduced, one into each naris, with the flat portion in contact with the septum on each side, and the curved wings in contact with the inferior turbinated bodies. When so applied they are firmly retained within the nares, and they so fix the septum in the middle line as to thoroughly support the fractured structures at a higher level. They have at least two points which render them much superior in practice to the intra-nasal splints or plugs recommended by Mr. Adams and Mr. Walsham: (1) From their weight and method of fixation they are not readily displaced—they remain fixed in any desired position; and (2) the arch formed by the wings ensures patency of the nares while the

Fig. 4.—Shape of Mask, half-size, to be moulded.
F Forehead portion. N Nose portion. A A Alar parts.

splints are being worn, a most important point for the patient's comfort.

The mask is then applied, having been carefully moulded to the desired form, and when adjusted it is fixed by strips of adhesive plaster. It can be readily modelled to fit accurately any form of nose, and by it pressure may be extended equally all over or on any particular portion of the organ.

When the deformity to be dealt with is the result of an old standing fracture, before attempting to rectify the deformity we must carefully consider the state of the patient's health as well as the form of the organ, as the treatment necessary is more or less severe. For the successful treatment of such cases the parts involved must, after the interior of the nose has been well cleansed, be thoroughly broken up, the mere twisting or cracking of one portion is never sufficient. So broken up the parts can be moulded at pleasure and placed in such positions as to restore the normal contour of the nose, or even to improve upon its original form.

At the end of four to six days the intra-nasal splints may be removed and not returned unless specially called for, and the external splint may be removed after six days, the parts inspected, and the splint re-applied. It is seldom necessary to have it worn for a longer time than two weeks in all.

## Figures and Tables

**Fig. 1. f1:**
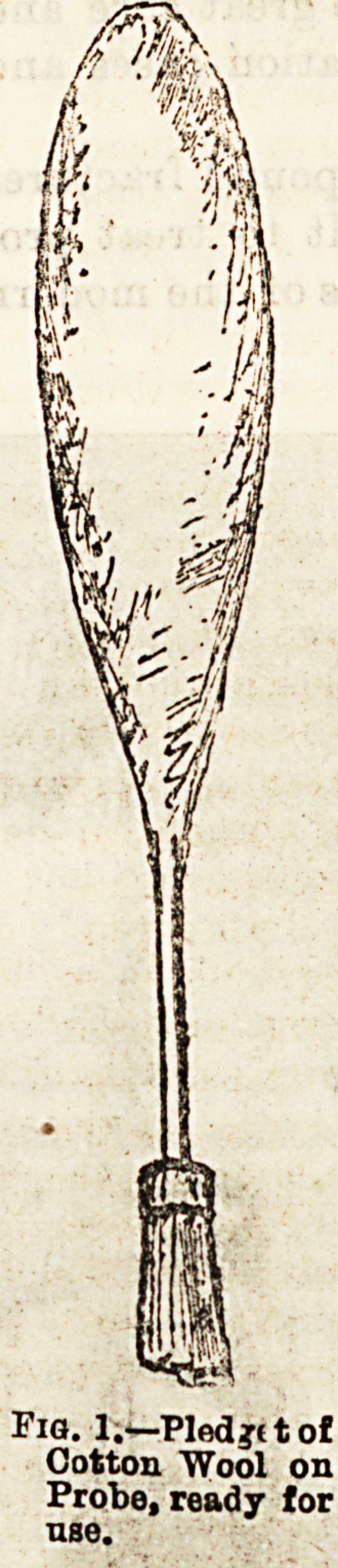


**Fig. 2. f2:**
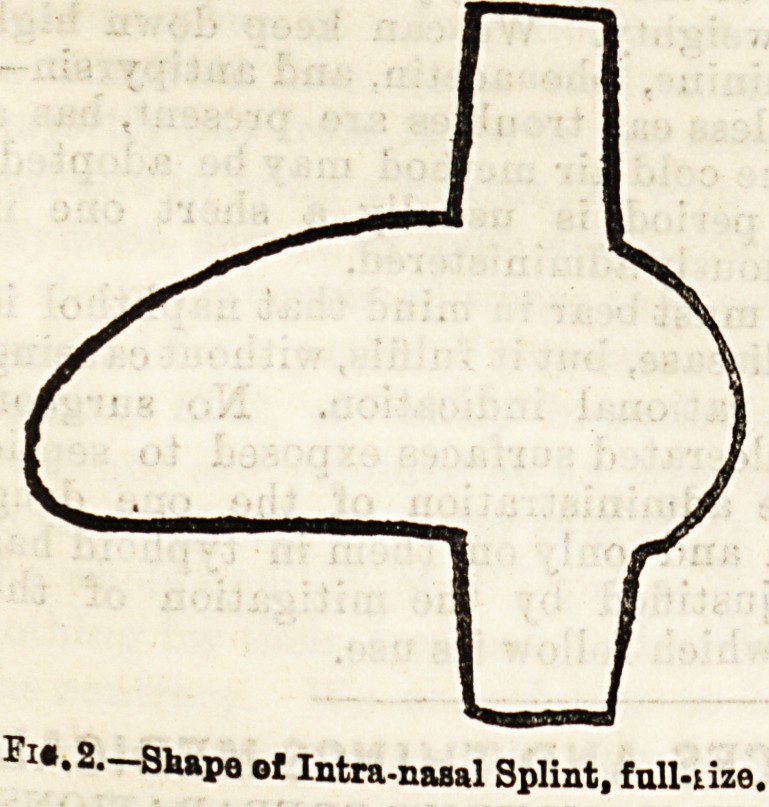


**Fig. 3. f3:**
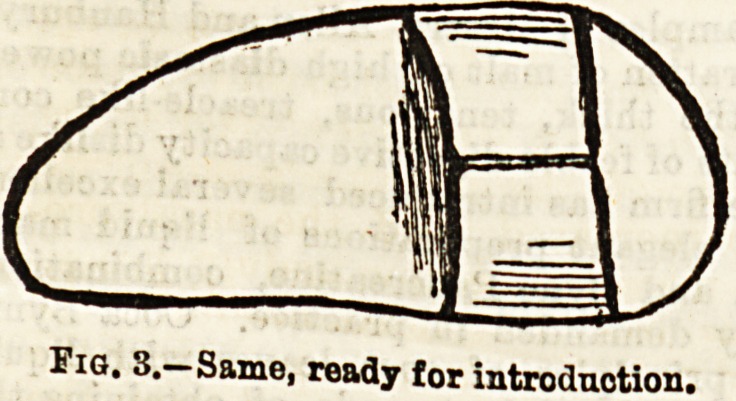


**Fig. 4. f4:**